# Simulation evaluation of statistical properties of methods for indirect and mixed treatment comparisons

**DOI:** 10.1186/1471-2288-12-138

**Published:** 2012-09-12

**Authors:** Fujian Song, Allan Clark, Max O Bachmann, Jim Maas

**Affiliations:** 1 Norwich Medical School, Faculty of Medicine and Health Science, University of East Anglia, Norwich, Norfolk, NR4 7TJ, UK

**Keywords:** Indirect comparison, Mixed treatment comparison, Network meta-analysis, Inconsistency, Bias, Type I error, Statistical power, Simulation evaluation

## Abstract

**Background:**

Indirect treatment comparison (ITC) and mixed treatment comparisons (MTC) have been increasingly used in network meta-analyses. This simulation study comprehensively investigated statistical properties and performances of commonly used ITC and MTC methods, including simple ITC (the Bucher method), frequentist and Bayesian MTC methods.

**Methods:**

A simple network of three sets of two-arm trials with a closed loop was simulated. Different simulation scenarios were based on different number of trials, assumed treatment effects, extent of heterogeneity, bias and inconsistency. The performance of the ITC and MTC methods was measured by the type I error, statistical power, observed bias and mean squared error (MSE).

**Results:**

When there are no biases in primary studies, all ITC and MTC methods investigated are on average unbiased. Depending on the extent and direction of biases in different sets of studies, ITC and MTC methods may be more or less biased than direct treatment comparisons (DTC). Of the methods investigated, the simple ITC method has the largest mean squared error (MSE). The DTC is superior to the ITC in terms of statistical power and MSE. Under the simulated circumstances in which there are no systematic biases and inconsistencies, the performances of MTC methods are generally better than the performance of the corresponding DTC methods. For inconsistency detection in network meta-analysis, the methods evaluated are on average unbiased. The statistical power of commonly used methods for detecting inconsistency is very low.

**Conclusions:**

The available methods for indirect and mixed treatment comparisons have different advantages and limitations, depending on whether data analysed satisfies underlying assumptions. To choose the most valid statistical methods for research synthesis, an appropriate assessment of primary studies included in evidence network is required.

## Background

Indirect and mixed treatment comparisons have been increasingly used in health technology assessment reviews [1-4]. Indirect treatment comparison (ITC) refers to a comparison of different treatments using data from separate studies, in contrast to a direct treatment comparison (DTC) within randomised controlled trials. Statistical methods have been developed to indirectly compare multiple treatments and to combine evidence from direct and indirect comparisons in mixed treatment comparison (MTC) or network meta-analysis [5-9].

The existing simple [5] or complex [6-8] statistical methods for ITC and MTC are theoretically valid if certain assumptions can be fulfilled [2,10]. The relevant assumptions could be specifically classified according to a conceptual framework that delineates the homogeneity assumption for conventional meta-analysis, the similarity assumption for adjusted ITC, and the consistency assumption for pooling direct and indirect estimates by MTC [2,11]. Among the basic assumptions, heterogeneity in meta-analysis and inconsistency between direct and indirect estimates can be quantitatively investigated. The presence of inconsistency between direct and indirect estimates has been empirically investigated in meta-epidemiological studies and numerous cases reports [12-16]. A range of statistical methods have been suggested to investigate the inconsistency in network meta-analysis [5,7,9,17-19].

The statistical properties of simple adjusted ITC [5] have been previously evaluated in simulation studies [1,20,21]. However, there are no simulation studies that formally evaluate methods for Bayesian network meta-analysis. In this simulation study, we comprehensively evaluated properties and the performance of commonly used ITC and MTC methods. Specifically, the objectives of the study are (1) to investigate bias, Type I error and statistical power of different comparison models for estimating relative treatment effects, and (2) to investigate bias, Type I error and statistical power of different comparison models for quantifying inconsistency between direct and indirect estimates.

## Methods

### Comparison models investigated

We investigated the performance of the following ITC and MTC statistical models.

#### Adjusted indirect treatment comparison (AITC)

This frequentist based method is also called as Bucher’s method [5], based on the assumption that indirect evidence is consistent with the direct comparison. Suppose that treatment A and B are compared in RCT-1 (with *d*_*AB*_ as its result, logOR for example), and treatment A and C compared in RCT-2 (with *d*_*AC*_as its result). Then treatment A can be used as a common comparator to adjust the indirect comparison of treatment B and C:

$$d_{\mathrm{BC}}^{\mathrm{Ind}}=d_{\mathrm{AB}}-d_{\mathrm{AC}}$$

Its variance is:

$$Var\left(d_{\mathrm{BC}}^{\mathrm{Ind}}\right)=Var\left(d_{\mathrm{AB}}\right)+Var\left(d_{\mathrm{AC}}\right)$$

When there are multiple trials that compared treatment A and B or treatment A and C, results from individual trials can be combined using fixed-effect or random-effects model. Then the pooled estimates of *d*_*AB*_ and *d*_*AC*_ are used in the AITC.

#### Consistency frequentist MTC (CFMTC)

The results of frequentist ITC (using the Bucher’s method) can be combined with the result of frequentist DTC in a MTC. The frequentist combination of the DTC and ITC estimate is weighted by the corresponding inverse of variance, as for pooling results from two individual studies in meta-analysis [22].

This MTC is termed ‘consistency MTC’, as it assumes that the result of direct comparison of treatment *B* and *C* statistically equals to the result of indirect comparison of *B* and *C* based on the common comparator *A*[9]. Suppose a network of three sets of trials that compared A vs. B, A vs. C, and B vs. C, we only need to estimate two basic parameters *d*_*AB*_ and *d*_*AC*_, and the third contrast (functional parameter) can be derived by *d*_*BC*_ = *d*_*AB*_ - *d*_*AC*_.

#### Consistency Bayesian MTC (CBMTC)

As the CFMTC, this model is also based on the assumption that ITC is consistent with DTC [8]. Suppose that several treatments (A, B, C, and so on) are compared in a network of trials. We need to select a treatment (treatment *A*, for example, placebo or control) as the *reference* treatment. In each study, we also consider a treatment as the *base* treatment (*b*). Below is the general model for the consistency MTC:

$$\theta_{\mathrm{kt}}=\{\begin{array}{c} \mu_{\mathrm{kb}}\;b=A,B,C,\;if\;t=b\\ \mu_{\mathrm{kb}}+\delta_{\mathrm{kbt}}\;t=B,C,D,\;if\;t\;is\;after\;b \end{array}$$

$$\delta_{\mathrm{kbt}}\sim N\left(d_{\mathrm{bt}},\tau^{2}\right)$$

$$d_{\mathrm{bt}}=d_{\mathrm{At}}-d_{\mathrm{Ab}}$$

$$d_{\mathrm{AA}}=0$$

Here *θ*_*kt*_ is the underlying outcome for treatment *t* in study *k*, *μ*_*kb*_ is the outcome of treatment *b*, and *δ*_*kbt*_ is the relative effect of treatment *t* as compared with treatment *b* in study *k*. The trial specific relative effect *δ*_*kbt*_ is assumed to have a normal distribution with a mean *d*_*bt*_ and variance τ^*2*^ (i.e., between study variance). When τ^*2*^ = 0, this model provides results as a fixed-effect analysis.

#### Random Inconsistency Bayesian MTC (RIBMTC)

Some authors assumed that inconsistencies (that is, the differences between *d*_*BC*_ from direct comparisons and $d_{\mathrm{BC}}^{\mathrm{Ind}}$ based on indirect comparison) have a common normal distribution with mean 0 and variance $\sigma_{\omega }^{2}$[7,9]. These methods have been termed the “random inconsistency model” [23]. In this study, we evaluated the random inconsistency model by Lu and Ades [9]. This model can be expressed by the following:

$$d_{\mathrm{BC}}=d_{\mathrm{AB}}-d_{\mathrm{AC}}+\omega_{\mathrm{BC}}\text{,}$$

and

$$\omega_{\mathrm{BC}}\sim N\left(0,\sigma_{\omega }^{2}\right)\text{.}$$

Here ω_BC_ is termed inconsistency factor (ICF).

#### Inconsistency Bayesian Meta-Analysis (IBMA)

In the inconsistency Bayesian meta-analysis (IBMA), each of the mean relative effects (*d*_*xy*_) is separately estimated without using indirect treatment comparison information. The IBMA analysis is equivalent to a series of pair-wise DTC meta-analyses, although a common between-study variance (τ^2^) across different contrasts is assumed [24].

We originally intended to include the Lumley’s frequentist method for network meta-analysis [7]. However, it was excluded because of convergence problems during computer simulations.

### Inconsistency test

Let *d*_*BC*_ denote the natural log OR estimated by the DTC, and $d_{\mathrm{BC}}^{\mathrm{Ind}}$denote the log OR estimated by the ITC. The inconsistency (ω_*BC*_) in the results between the direct and indirect comparison of treatment *B* and *C* can be calculated by the following:

$$\omega_{\mathrm{BC}}=d_{\mathrm{BC}}-d_{\mathrm{BC}}^{\mathrm{Ind}}$$

When the estimated ω_*BC*_ is greater than 0, it indicates that the treatment effect is over-estimated by the ITC as compared with the DTC. For Bucher’s method [5,12], the calculation of inconsistency was based on the pooled estimates of *d*_*BC*_ and $d_{\mathrm{BC}}^{\mathrm{Ind}}$ by meta-analyses. The variance of the estimated inconsistency was calculated by:

$$Var\left(\omega_{\mathrm{BC}}\right)=Var\left(d_{\mathrm{BC}}\right)+Var\left(d_{\mathrm{BC}}^{\mathrm{Ind}}\right)$$

where Var(*d*_*BC*_) and Var( $d_{\mathrm{BC}}^{\mathrm{Ind}}$) are the variance of *d*_*BC*_ and $d_{\mathrm{BC}}^{\mathrm{Ind}}$respectively. The null hypothesis that the DTC estimate equals to the ITC estimate was tested by *Z* statistic

$$Z_{\mathrm{BC}}=\frac{\omega_{\mathrm{BC}}}{\sqrt{Var\left(\omega_{\mathrm{BC}}\right)}}$$

If the absolute value of *Z*_*BC*_ is greater than 1.96, the observed inconsistency is considered to be statistically significantly different from zero.

The estimate of inconsistency is not applicable when the consistency Bayesian MTC model [8] is used. With the inconsistency Bayesian meta-analysis (IBMA), the estimate of *d*_*BC*_ is naturally available, and $d_{\mathrm{BC}}^{\mathrm{Ind}}$ can be easily estimated based on *d*_*AB*_ and *d*_*AC*_, as by the “node-splitting” method [17,24]. The point estimate of inconsistency in Bayesian MTC was the average (mean value) of the simulated results. The significance of the inconsistency was based on the estimated 95% intervals. If the 95% intervals did not contain the zero, the observed inconsistency was considered to be statistically significant.

The random inconsistency Bayesian MTC (RIBMTC) model assumes that the inconsistency within a network of trials is normally distributed with mean ω = 0 and variance $\sigma_{\omega }^{2}$[9]. We also recorded the estimated ω and $\sigma_{\omega }^{2}$ by the RIBMTC model.

### Simulation scenarios

**Figure 1:**
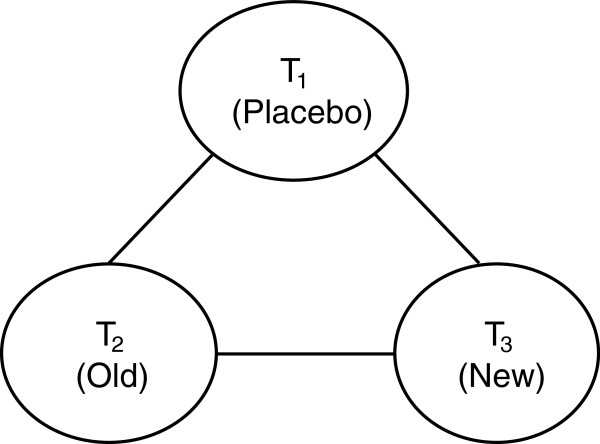
Network of simulated trials.

In this study, a simple network of two-arm trials with a closed loop was simulated to separately compare three treatments: treatment 1 (T_1_, placebo), treatment 2 (T_2_, an old drug), and treatment 3 (T_3_, a new drug) (Figure 1). The comparison of T_2_ and T_3_ was considered as the main interest. Trials that compared T_1_ vs. T_2_ and trials that compared T_1_ vs. T_3_ were used for the indirect comparison of T_2_ and T_3_. Given the available resource, a limited number of simulation scenarios were adopted in this study. The following simulation parameters were decided after considering characteristics of published meta-analyses (also see Table 1).

• The number of patients in each arm of a pair-wise trial is 100. The number of trials for each of the three contrasts is 1, 5, 10, 20, 30 and 40. A scenario of imbalanced number of trials (including a single trial for one of the three sets) is also included.

We use odds ratio (OR) to measure the outcome [25]. The assumed true OR_12_ = 0.8, and the true OR_13_ = 0.8 or 0.6. When OR is less than 1 (or *log OR < 0*), it indicates that the risk of events is reduced by the second of the two treatments compared.

• The true $\log OR_{23}$ is calculated by: $\log OR_{23}=\log OR_{13}-\log OR_{12}\text{.}$

• The baseline risk in the control arm is assumed to be 20% or 10%.

• It is assumed that heterogeneity is constant across different comparisons, and there are four levels of between study variance: *τ*^*2*^ = 0.00, 0.05, 0.10, and 0.15 respectively [26].

• The trial-specific natural log OR (*d*_*kij*_) in study *k* used to generate simulated trials is based on the assumed true log OR and the between-trial variance: $d_{\mathrm{kij}}\sim N\left(d_{\mathrm{ij}},\tau^{2}\right)\text{.}$

• Given the baseline risk (*P*_*k*1_) and the trial-specific OR, the risk in the treatment arm in study *k* is calculated by:

•

$$P_{\mathrm{kt}}=\frac{P_{k1}\times Exp\left(d_{k1t}\right)}{1-P_{k1}+P_{k1}\times Exp\left(d_{k1t}\right)}\text{.}$$

• Bias in a clinical trial can be defined as a systematic difference between the estimated effect size and the true effect size [27]. It is assumed here that all bias, where it exists, will result in an over-estimated treatment effect of active drugs (T_2_ and T_3_) as compared with placebo (T_1_), and an over-estimated treatment effect of the new drug (T_3_) relative to the old drug (T_2_). The extent of bias and inconsistency is measured by ratio of odds ratios (ROR). When ROR = 1, it indicates that there is no bias. When ROR = 0.8, it means that the effect (OR) of a treatment is over-estimated by 20%.

**Table 1:** Simulation input parameters

**Parameters**	**Values**
Number of studies	3×40; 3×20; 3×10; 3×5; 3×1; 5/1/5
Number of patients per study	2×100
Between trial heterogeneity: τ^2^	0.00; 0.05; 0.10; 0.15
Treatment effect: log OR, θ_12_	log(0.8)
Treatment effect: log OR, θ_13_	log(0.8); log(0.6)
Bias: ROR_12_	0.00; 0.80
Bias: ROR_13_	0.00; 0.80
Bias: ROR_23_	0.00; 0.80
Baseline risk: P_1_	10%; 20%

(Note: these input values could be combined differently for a large number of possible simulation scenarios).

A network of trials was randomly generated, using assumed input parameters (Table 1). For each arm of the simulated trial, the number of events was randomly generated according to the binomial distribution:

$$r_{\mathrm{ki}}\sim Binomial\left(N_{\mathrm{ki}},P_{\mathrm{ki}}\right)$$

Here, *N*_*ki*_ is the number of patients in the arm of treatment *i*, and *P*_*ki*_ is the risk of events given treatment *i* in study *k*. If the simulated number of events is zero, we added 0.5 to the corresponding cells of the 2x2 table for conducting inverse variance weighted meta-analysis.

### Data analysis

AITC and MTC were conducted using data from the simulated trials by fixed-effect and random-effects meta-analyses. For frequentist ITC, we used inverse variance weights to pool results of multiple trials in meta-analysis, and used the DerSimonian-Laird method for random-effects meta-analyses [22].

The performance of the ITC and MTC methods was measured by the type I error rate or statistical power, observed bias and mean squared error (MSE). We estimated the rate of type I error (when the null hypothesis is true) and the statistical power (when the null hypothesis is false) by the proportion of significant estimates (two sided α < 0.05) for the frequentist methods, or the proportion of estimates with a 95% interval that did not contain the zero treatment effect for the Bayesian methods.

We generated 5000 simulated results for each of the simulation scenarios in Table 1, and calculated the bias and mean squared error (MSE) as:

$$Bias(\overset{⌢}{\theta })=\frac{1}{5000}\sum_{c=1}^{5000}({\overset{⌢}{\theta }}_{c}-\theta )$$

$$MSE(\overset{⌢}{\theta })=\frac{1}{5000}\sum_{c=1}^{5000}{\left({\overset{⌢}{\theta }}_{c}-\theta \right)}^{2}$$

where *ϑ* is the true parameter value, $\overset{⌢}{\theta }$_c_ is the estimated value from the c^th^ simulated data set. Monte Carlo 95% intervals for estimated mean bias and inconsistency were based on the 2.5% and 97.5% percentiles of the corresponding estimates.

### Computing implementation

Bayesian network meta-analyses were implemented by Markov chain Monte Carlo (MCMC) methodology [8]. Vague or non-informative priors were used for MCMC simulations. Each simulation comprised 20,000 ‘burn-in’ iterations followed by 40,000 posterior mean sample iterations. Posterior mean samples collected were thinned by a ratio of 5:1 to resulting in 8,000 final posterior mean samples from each MCMC simulation. We used R 2.13.0 [28] and related packages (RJAGS) to generate data and to sample Bayesian posterior distributions. All simulations were carried out on the High Performance Computing Cluster supported by the Research Computing Service at the University of East Anglia.

## Results

For the purpose of simplification, we only presented the results of selected representative scenarios below.

### Estimating relative treatment effects

#### MSE and bias

**Figure 2:**
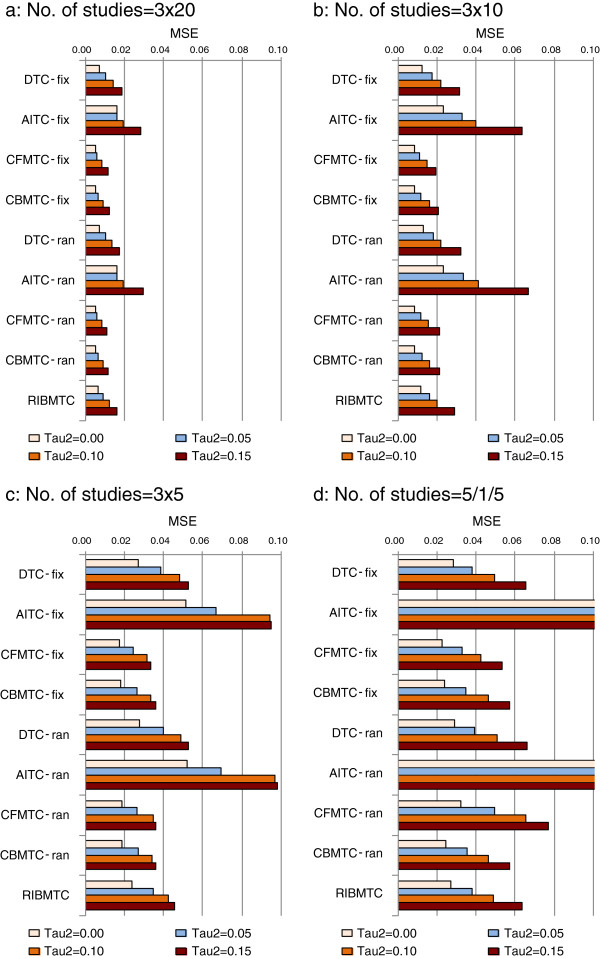
**Mean squared error (MSE) by different comparison models (Note: baseline risk 20%; zero treatment effect; without systematic bias in trials; fix, fixed effect; ran, random-effects; Tau2 refers τ**^**2**^**).**

As expected, mean squared error (MSE) is positively associated with the small number of studies, and large heterogeneity in meta-analysis (Figure 2). Of the comparison methods investigated, the AITC method has the largest MSE. With the existence of heterogeneity, there are no noticeable differences in MSE between the fixed-effect and random-effects models.

**Figure 3:**
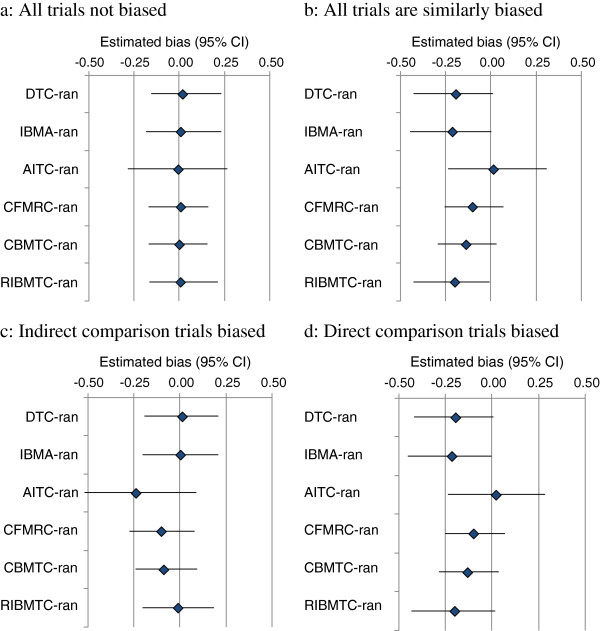
**Bias by different comparison methods (Note: selected simulation scenarios, baseline risk = 20%; τ**^**2**^ **= 0.05; number of studies =3x20; random-effects analyses).**

When there is no bias in simulated trials, the results of the all comparison methods are on average unbiased (Figure 3a). When all trials are similarly biased, the DTC and the inconsistency Bayesian MTC (RIBMTC) are fully biased, while the AITC is not biased (Figure 3b). When only the trials involved in AITC are biased, the DTC and inconsistency MTC models are unbiased (Figure 3c). The extent of bias in the consistency MTC models (both CFMTC and CBMTC) lies between the DTC and ITC. The impacts of biases in primary studies on the validity of different comparison methods are summarised in Table 2.

**Table 2:** Impact of simulated biases on the results of different comparison methods

**Comparison methods**	**Actual true biases**			
	**Trials not biased**	**All trials similarly biased**	**One set of AIC trials biased**	**DC trials biased**
**Direct comparison (DTC)**	Not biased	Fully biased	Not biased	Fully biased
**Indirect comparison (AITC)**	Not biased	Not biased	Fully biased	Not biased
**Consistency frequentist MTC**	Not biased	Moderately biased	Moderately biased	Moderately biased
**Consistency Bayesian MTC**	Not biased	Moderately biased	Moderately biased	Moderately biased
**Inconsistency Bayesian meta-analysis**	Not biased	Fully biased	Not biased	Fully biased
**Random inconsistency Bayesian MTC (RIBMTC)**	Not biased	Fully biased	Not biased	Fully biased

(Note: “Fully biased” – the bias equals the bias in trials; “Moderately biased” – as a result of combining biased direct estimate and unbiased indirect estimate, or a result of combining unbiased direct estimate and biased indirect estimate).

#### Type I error

**Figure 4:**
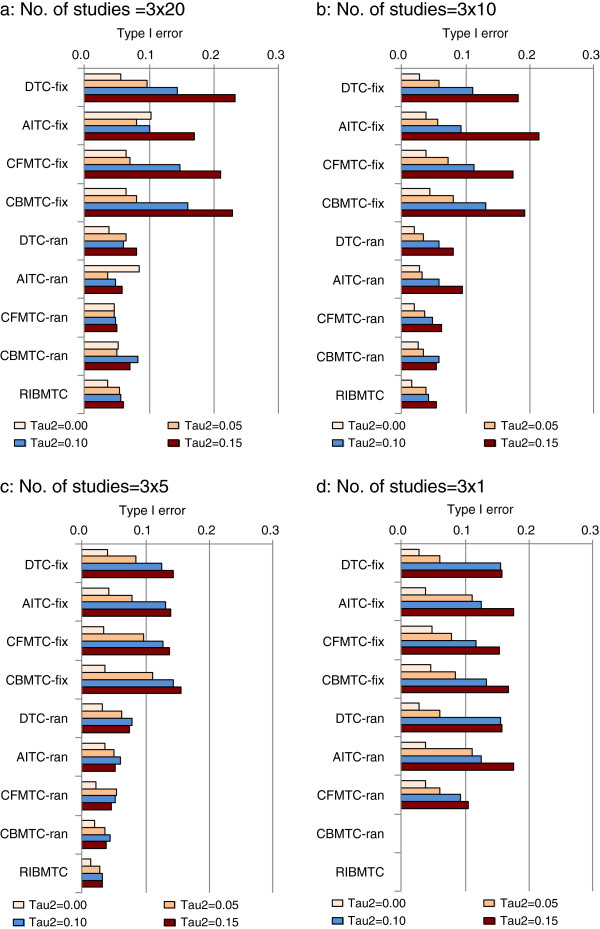
**Type I error – proportion of significant results when true treatment effect is zero, impact of number of studies and assumed heterogeneity (Note: baseline risk =20%; fix, fixed effect; ran, random-effects; Tau2 refers τ**^**2**^**).**

Assuming zero heterogeneity across studies, there are no clear differences in the rate of type I error between different MTC methods (Figure 4). The extent of heterogeneity was clearly associated with inflated rates of type I error. In the presence of great heterogeneity, the rate of type I error is particularly large when fixed-effect models are applied. The random-effects models tend to have values closer to 0.05. However, random-effects models no longer have advantages when there is only a single study available for each of the three comparisons (Figure 4d). When there is only a single study for each of the three contrasts, the rate of type I error is zero by Bayesian random-effects models (CBMTC and RIBMTC), which seems due to the unchanged vague or non-informative priors [26]. Within the fixed-effect models the different methods have similar type I error rates, as well as within the random-effects models (Figure 4).

As expected, the higher baseline risk (20%) is associated with the higher rate of type I error as compared with the lower baseline risk (10%) (data not shown).

#### Statistical power

**Figure 5:**
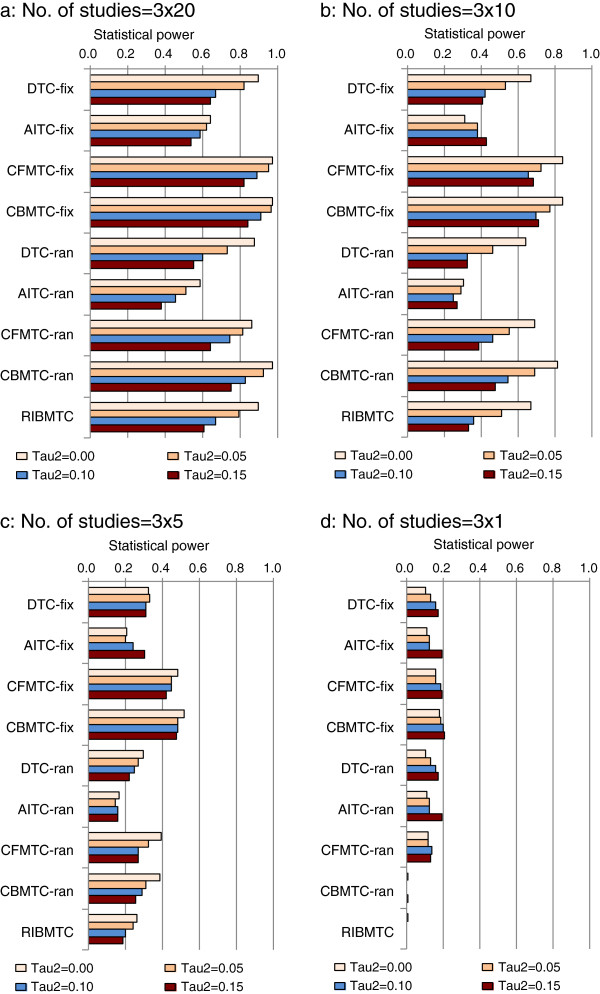
**Statistical power to detect treatment effect (OR23 = 0.75), impact of number of studies and assumed heterogeneity (Note: Baseline risk =20%; fix, fixed effect; ran, random-effects; Tau2 refers τ**^**2**^**).**

As expected, the statistical power (*1-β*) is positively associated with the number of studies (Figure 5). As compared with the DTC, the statistical power of AITC is low. The pooling of DTC and AITC evidence in MTC increases the statistical power (Figure 5).

With a larger number of studies, the statistical power of all methods is reduced by the presence of heterogeneity (Figure 5a-b). The association between heterogeneity and statistical power becomes unclear when the number of studies is small (Figure 5c-d). When there is only a single study, the statistical power of all the methods is extremely low, and it is zero by the Bayesian random-effects models (again, due to vague or non-informative priors) (Figure 5d).

A expected, the statistical power is reduced when the baseline risk is lowered from 20% to 10% (data no shown).

### Inconsistency detecting

**Figure 6:**
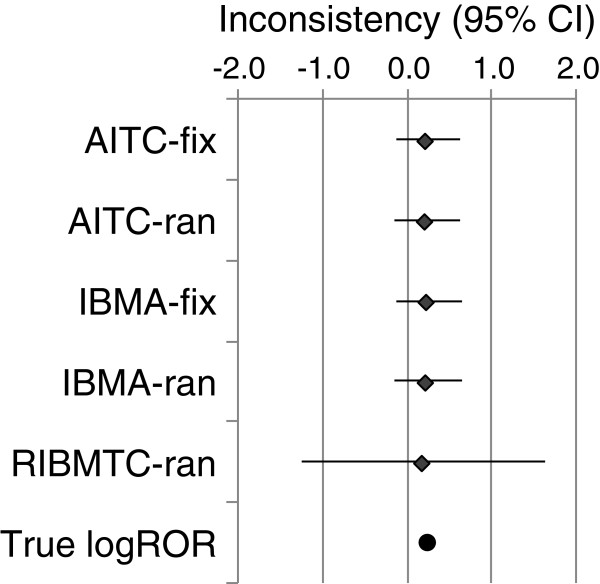
**Estimated inconsistency (log ROR) – a selected simulation scenario (Note: true logROR = 0.223; baseline risk = 20%; number of studies =3x20; τ**^**2**^ **= 0.10; Tau2 refers τ**^**2**^**).**

The estimated inconsistencies by the different comparison methods are on average unbiased, but the 95% intervals are wide (Figure 6). The 95% interval of the estimated inconsistency by the RIBMTC method is much wider than by other methods.

**Figure 7:**
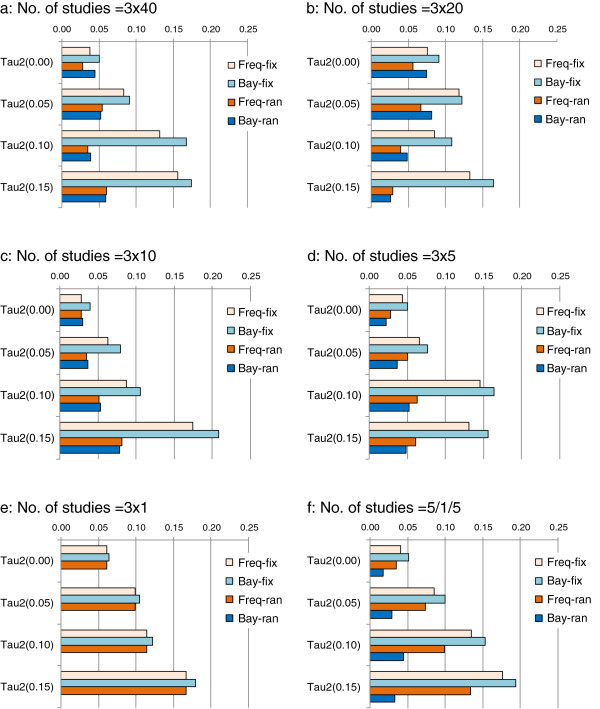
**Type I error for inconsistency detection: impact of heterogeneity and number of studies (Note: baseline risk =20%, true lnROR = 0; tau2 refers τ**^**2**^**; Freq-fix, frequentist fixed-effect; Freq-ran, frequentist random-effects; Bay-fix, Bayesian fixed-effect; Bay-ran, Bayesian random-effects).**

Heterogeneity is positively associated with the rate of type I error for detecting inconsistency by the fixed-effect models, while the number of studies does not noticeably affect the rate of type I error (Figure 7). However, when there is only a single study for each of the three contrasts, the Bayesian random-effects method has zero type I error (due to the vague or non-informative priors for τ), and the rate of type I error by frequentist random-effects model was similar to the fixed-effect models (Figure 7e). When there is imbalanced and singleton number of trials, the frequentist random-effects model has larger type I errors than the Bayesian random-effects method (Figure 7f).

**Figure 8:**
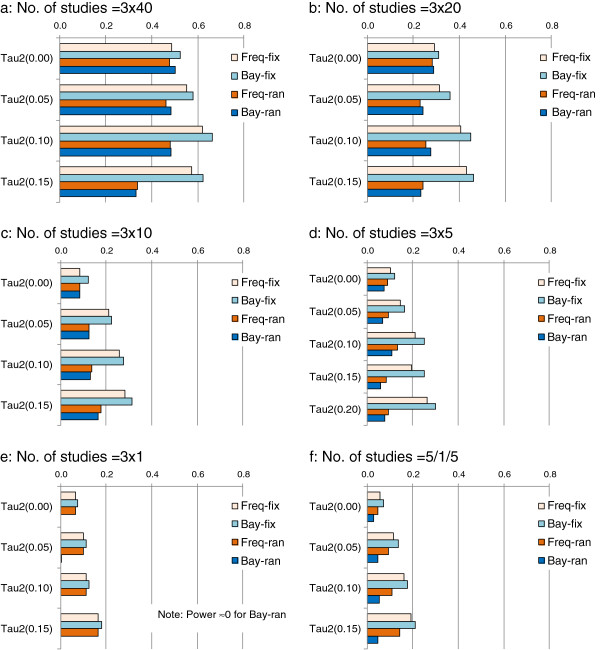
**Statistical power to detect inconsistency: impact of heterogeneity and number of studies (Note: baseline risk =20%, true lnROR = 0.223; tau2 refers τ**^**2**^**. Freq-fix, frequentist fixed-effect; Freq-ran, frequentist random-effects; Bay-fix, Bayesian fixed-effect; Bay-ran, Bayesian random-effects).**

The statistical power to detect the specified inconsistency (P < 0.05) increases with the increasing number of studies (Figure 8). However, the statistical power is still lower than 70% even when there are 120 studies (200 patients in each study) in the trial network (Figure 8a). By fixed-effect model, the existence of heterogeneity generally increases the power to detect inconsistency. However, the impact of heterogeneity on the power of random-effects models is unclear. When there is only one study for each of the three contrasts, the power by Bayesian random-effects model is about zero (given vague or non-informative priors for τ^2^) (Figure 8e).

## Discussion

### Summary of findings

Mean squared error (MSE) reflects a combination of both bias and random error, which is clearly associated with the number of studies, heterogeneity, and the baseline risk. When simulated studies are not biased, the AITC method had the largest MSE, as compared with DTC and MTC methods. Given the same comparison approach, there are no noticeable differences in estimated MSE between the fixed-effect and random-effects models.

When simulated trials are unbiased, the results of all comparison methods investigated are good at predicting the true magnitude and direction of the effect. However, there are simulation scenarios under which AITC could be biased. When all trials are similarly biased, the results of AITC will be less biased than the results of DTC. This finding is consistent with the result of a previous study that evaluated the impacts of biases in trials involved in AITC [29]. Bias by MTC will lie between the bias by DTC and AITC (Table 2).

It should be noted that, in addition to the scenarios simulated in this study, bias in original trials may also be magnified if the two sets of trials for the AITC are biased in opposite directions. For example, it is possible that the relative effect of a treatment versus the common comparator is over-estimated in one set of trials, and under-estimated in another set of trials. Under this circumstance, the AITC estimate will be biased and the extent of such bias will be greater than the extent of bias in the original studies.

#### Estimating comparative treatment effect

The type I error of ITC and MTC methods are associated with the extent of heterogeneity, whether a fixed-effect or random-effects meta-analysis is used, and the level of baseline risk. There are no noticeable differences in type I error between different comparison methods.

As expected, the number of studies is clearly associated with the statistical power to detect specified true treatment effect. The AITC method has the lowest statistical power. When there is no assumed inconsistency or bias, the MTC increases the statistical power as compared with the power of DTC alone. There are no noticeable differences in the statistical power between different MTC methods.

#### Inconsistency testing

We found that the all comparison methods are on average unbiased for estimating the inconsistency between the direct and indirect estimates. The 95% intervals by the RIBMTC method are much wider than that by other methods. Heterogeneity inflates the type I error in the detection of inconsistencies by fixed-effect models. When there are singleton studies in the trial network, the frequentist based random-effects model has relatively larger type I error than the Bayesian random-effects model.

As expected, the power to detect inconsistency is positively associated with the number of studies and the use of fixed-effect models. For the inconsistency detection, heterogeneity increases the power of fixed-effect models, but reduces the power of random-effects models when the number of studies is large.

### Comparing with previous studies

Methods of frequentist based indirect comparison have been investigated in several previous simulation studies [1,20,21]. A study found that the Bucher’s method and logistic regression generally provided unbiased estimates [1]. The simulation scenarios evaluated in that study was limited by using data from a single trial. In another study, Wells and colleagues simulated variance, bias and MSE by the DTC and AITC method [21]. It was reported that the observed variance, bias and MSE for the AITC were larger than that for the DTC, particularly when the baseline risk was low [21]. A more recent simulation study by Mills and colleagues reported findings from an investigation of the Bucher’s ITC method [20]. They found that the AITC method lacks statistical power, particularly in the presence of heterogeneity, and has high risk of over-estimation when only a single trial is available in one of the two trial sets. However, they did not compare the performance of the AITC and the corresponding DTC or MTC [20].

Bayesian MTC methods have not been investigated in previous simulation studies. In the current study, we investigated the performance of statistical methods for DTC, AITC, frequentist and Bayesian MTC. The simulation results reveal the complex impacts of biases in primary studies on the results of direct, indirect and mixed treatment comparisons. When the simulated primary studies are not systematically biased, the AITC and MTC methods are not systematically biased, although the AITC method has the largest MSE. Depending on the extent and direction of bias in primary studies, the AITC and MTC estimates could be more or less biased than the DTC estimates.

In the existence of heterogeneity and a small number of studies, AITC and MTC methods have indeed the inflated rate of type I error and a low statistical power. It is important to note that the performance of the corresponding DTC is similarly affected. The performance of the DTC method is superior to the performance of the AITC method. However, the statistical power of MTC is generally higher than the corresponding DTC.

It is the first time that the power to detect inconsistency in network meta-analysis has been investigated by simulations. The low power to detect inconsistency in network meta-analysis seems similar to the low power to detect heterogeneity in pair-wise meta-analysis [30].

### Limitations of the study

Due to the restriction of available resource, a limited number of simulation scenarios were considered. Clearly, the performance of a model will depend on whether the simulation scenario matches the model’s assumptions. For example, the fixed-effect model should not be used when there is heterogeneity across multiple studies, in order to avoid the inflated type I error.

In this paper, the simple network containing three sets of two-arm trials with a single completed loop is considered. We evaluated the methods for detecting inconsistency, and did not consider models for investigating causes of inconsistency. Therefore, further simulation studies are required to evaluate complicated networks involving more than three different treatments and containing trials with multiple arms. In addition, further simulation studies are required to evaluate the performance of regression models that incorporate study-level covariates for investigating the causes of heterogeneity and inconsistency in network meta-analysis [18,19,31].

For MCMC simulations, we used vague or non-informative priors [32]. When the number of studies involved is large, finding of the study were unlikely to be different if more informative priors had been used. However, further research is required to investigate whether an informed prior for between-study variance would be more appropriate when the number of studies involved in a Bayesian meta-analysis is very small [26].

### Implications to practice and research

The results of any comparison methods (including direct comparison trials) may be biased as a consequence of bias in primary trials involved. To decide which comparison method may provide more valid or less biased results, it is helpful if we can estimate the extent and direction of possible biases in primary studies. Empirical evidence indicated the existence of bias in randomised controlled trials [33-35], particularly in trials that had outcomes subjectively measured without appropriate blinding [36,37]. Although it is usually difficult to estimate the magnitude of bias, the likely direction of bias may be estimated. For example, it may be assumed that possible bias was likely to result in an over-estimation of treatment effect of active or new drugs when they are compared with placebo or old drugs [38]. More complicated models could also be explored for estimating bias in evidence synthesis [39-41].

For detecting inconsistency, the fixed-effect methods have a higher rate of type I errors as well as a higher statistical power as compared with the random-effects methods. The performances of the Bayesian and frequentist methods are generally similar. When there are singleton trials in evidence network, the rate of type I error by frequentist random-effects method is larger than by the Bayesian random-effects method. This is due to the under-estimation of between-study variance by the frequentist method, while the Bayesian method provides an estimate of between-study variance using all data available in the whole network of trials [32]. However, when there is a single study for each of the all comparisons, Bayesian random-effects models should be avoided.

Imbalanced distribution of effect-modifiers across studies may be a common cause of both heterogeneity in pair-wise meta-analysis and evidence inconsistency in network meta-analysis [17]. However, it is helpful to distinguish the heterogeneity in pair-wise meta-analysis and inconsistency in network meta-analysis. Under the assumption of exchangeability, the results of direct and indirect comparisons could be consistent in the presence of large heterogeneity in meta-analyses. For example, the inflated type I error rate in detecting inconsistency by the fixed-effect models can be corrected by the use of random-effects models. It is also possible to observe significant inconsistencies between direct and indirect estimates when there is no significant heterogeneity in the corresponding pair-wise meta-analyses. The association between heterogeneity and the statistical power to detect inconsistency is complex, depending on whether the fixed-effect or random-effects model is used and the number of studies involved.

A major concern is the very low power of commonly used methods to detect inconsistency in network meta-analysis when it does exist. Therefore, inconsistency in network meta-analysis should not be ruled out based only on the statistically non-significant result of a statistical test. For all network meta-analysis, trial similarity and evidence consistency should be carefully examined [2,42].

## Conclusions

Of the comparison methods investigated, the indirect comparison has the largest mean squared error and thus the lowest certainty. The direct comparison is superior to the indirect comparison in terms of statistical power and mean squared error. Under the simulated circumstances in which there are no systematic biases and inconsistencies, the performances of mixed treatment comparisons are generally better than the performance of the corresponding direct comparisons.

When there are no systematic biases in primary studies, all methods investigated are on average unbiased. Depending on the extent and direction of biases in different sets of studies, indirect and mixed treatment comparisons may be more or less biased than the direct comparisons. For inconsistency detection in network meta-analysis, the methods evaluated are on average unbiased. The statistical power of commonly used methods for detecting inconsistency in network meta-analysis is low.

In summary, the statistical methods investigated in this study have different advantages and limitations, depending on whether data analysed satisfies the different assumptions underlying these methods. To choose the most valid statistical methods for network meta-analysis, an appropriate assessment of primary studies included in the evidence network is essential.

## Abbreviations

AITC: Adjusted indirect treatment comparison; CBMTC: Consistency Bayesian mixed treatment comparison; CFMTC: Consistency frequentist mixed treatment comparison; DTC: Direct treatment comparison; IBMA: Inconsistency Bayesian meta-analysis; ITC: Indirect treatment comparison; MCMC: Markov chain Monte Carlo; MSE: Mean squared error; MTC: Mixed treatment comparison; OR: Odds ratio; RCT: Randomised controlled trial; ROR: Ratio of odds ratios; RIBMTC: Random inconsistency Bayesian mixed treatment comparison.

## Competing interests

The authors declare that they have no competing interests.

## Authors’ contributions

FS, AC and MOB conceived the idea and designed research protocol. JM, AC and FS developed simulation programmes and conducted computer simulations. FS analysed data and prepared the draft manuscript. All authors commented on the manuscript. FS had full access to all the data in the study and takes responsibility for the integrity of the data and the accuracy of the data analysis.

## Pre-publication history

The pre-publication history for this paper can be accessed here:

http://www.biomedcentral.com/1471-2288/12/138/prepub
